# Berberine Mitigates Sepsis-Associated Acute Kidney Injury in Aged Rats by Preserving Mitochondrial Integrity and Inhibiting TLR4/NF-κB and NLRP3 Inflammasome Activations

**DOI:** 10.3390/antiox13111398

**Published:** 2024-11-15

**Authors:** Ruedeemars Yubolphan, Anongporn Kobroob, Apisek Kongkaew, Natthakarn Chiranthanut, Natthanicha Jinadang, Orawan Wongmekiat

**Affiliations:** 1 Department of Pharmacology, Faculty of Medicine, Chiang Mai University, Chiang Mai 50200, Thailand; ruedeemars.yub@cmu.ac.th (R.Y.); natthakarn.c@cmu.ac.th (N.C.); 2 Division of Physiology, School of Medical Sciences, University of Phayao, Phayao 56000, Thailand; anongporn.ko@up.ac.th; 3 Research Administration Section, Faculty of Medicine, Chiang Mai University, Chiang Mai 50200, Thailand; aphisek.k@cmu.ac.th (A.K.); natthanicha.k@cmu.ac.th (N.J.); 4 Integrative Renal Research Unit, Department of Physiology, Faculty of Medicine, Chiang Mai University, Chiang Mai 50200, Thailand

**Keywords:** acute kidney injury, sepsis, aging, berberine, oxidative stress, inflammation, mitochondria, TLR4/NF-κB signaling, NLRP3 inflammasome

## Abstract

Sepsis-associated acute kidney injury (SA-AKI) presents a severe challenge in the elderly due to increasing incidence, high mortality, and the lack of specific effective treatments. Exploring novel and secure preventive and/or therapeutic approaches is critical and urgent. Berberine (BBR), an isoquinoline alkaloid with anti-inflammatory, antioxidant, and immunomodulatory properties, has shown beneficial effects in various kidney diseases. This study examined whether BBR could protect against SA-AKI in aged rats. Sepsis was induced in 26-month-old male Wistar rats by cecal ligation and puncture (CLP), either with or without BBR pretreatment. CLP induction led to SA-AKI, as indicated by elevated serum levels of malondialdehyde, tumor necrosis factor-alpha, urea nitrogen, creatinine, and neutrophil gelatinase-associated lipocalin (NGAL), along with histopathological features of kidney damage. Key indicators of kidney oxidative stress, mitochondrial dysfunction, apoptosis, and activations of the Toll-like receptor 4/nuclear factor-kappa B (TLR4/NF-κB) signaling, including the nucleotide-binding domain, leucine-rich-containing family, and pyrin domain-containing-3 (NLRP3) inflammasome pathway, were also elevated following CLP induction. BBR pretreatment substantially mitigated these adverse effects, suggesting that it protects against SA-AKI in aged rats by reducing oxidative stress, preserving mitochondrial integrity, and inhibiting key inflammatory pathways. These findings highlight the potential of BBR as a therapeutic agent for managing SA-AKI in elderly populations.

## 1. Introduction

Sepsis poses a life-threatening condition due to the body’s exaggerated response to the infection, leading to widespread inflammation, which can result in multiorgan dysfunction and failure [1]. Acute kidney injury (AKI) is one of the most common and severe comorbidities of sepsis, accounting for 60% of patients with sepsis [1]. Sepsis-associated AKI (SA-AKI) is a complex and serious clinical syndrome that involves multiple factors and is associated with high mortality. The incidence of SA-AKI significantly increases with age, with a mortality rate of around 50% in older patients. This rate continues to rise with advancing age, especially in those with pre-existing health issues, due to declines in immune and kidney function associated with aging [2]. SA-AKI in elderly patients regularly necessitates prolonged hospital stays, increases healthcare costs, and, most importantly, is correlated with a high mortality rate. Currently, there are limited treatment options available for elderly patients with SA-AKI, and traditional treatments such as fluid replacement and supportive care are not sufficiently effective in reducing mortality [2]. In addition, SA-AKI remains a critical challenge in elderly patients who survive an AKI episode. Evidence has shown that patients older than 65 years have an approximately 30% higher risk of non-fully recovering renal function after SA-AKI incidence, while 15–20% of these patients progress unrelentingly to chronic kidney disease and, eventually, end-stage renal disease [3]. Therefore, there is an urgent need to explore new approaches to prevent or reduce the severity of SA-AKI in this vulnerable population.

Oxidative stress, inflammation, mitochondrial dysfunction, and apoptosis have all been reported to contribute to the pathophysiology of sepsis, AKI, and aging [1,4,5]. Understanding and impeding the interplay between these mediators is crucial for developing effective prevention and treatment strategies to mitigate the severe impact of SA-AKI. Berberine (BBR) is a natural isoquinoline alkaloid derived from various medicinal plants such as *Berberis vulgaris* and *Coptis chinensis*. It possesses a diverse range of pharmacological properties, including anti-inflammatory, antioxidant, and immunomodulatory actions [6,7]. Currently, BBR plays a role in treating various diseases such as cardiovascular diseases, cognitive impairment, and diabetes, and it is also utilized as an antibacterial agent [8,9]. Importantly, BBR has a broad safety margin when administered orally [9].

Focusing on the kidney, studies have found that BBR has promising renoprotective effects against different renal conditions, including diabetic nephropathy, renal fibrosis, renal ischemia, renal stones, and renal aging [9]. It also protects against nephrotoxicity induced by chemotherapy, heavy metals, aminoglycosides, non-steroidal anti-inflammatory drugs (NSAIDs), and others [8,9,10,11,12]. The beneficial outcomes of BBR have been demonstrated through mechanisms such as upregulating mitophagy and utilizing its antioxidant, anti-inflammatory, and antiapoptotic properties [8,9,10,11,12]. These effects imply that BBR has evolving therapeutic potential against acute renal failure and chronic renal diseases. However, the role of BBR in sepsis-associated AKI, particularly in aging, has never been addressed.

Due to the increasing prevalence of infections in the geriatric population and the lack of effective treatment options for AKI caused by sepsis, this research aimed to evaluate the preventive effects of BBR as well as the key molecular mechanisms involved in such a context using a natural aging rat model of sepsis induced by cecal ligation and puncture (CLP). Molecular-level insights gained from this study could provide new knowledge about the effects and mechanisms of BBR in preventing SA-AKI in the elderly. This may lead to the development of new and more effective strategies for preventing and treating renal complications in elderly individuals with infections, ultimately reducing morbidity and mortality and improving the quality of life in this vulnerable population.

## 2. Materials and Methods

### 2.1. Chemicals, Reagents, and Antibodies

Berberine chloride (BBR, cat. no. B3251) and other analytical-grade chemicals and reagents used in this study (unless otherwise specified) were purchased from Sigma-Aldrich^®^ (Merck KGaA, Darmstadt, Germany). Antibodies against tumor necrosis factor-receptor (TNF-R, cat. no. 13377), phospho-NFκB p65 (pNF-κB, cat. no. 3033), nucleotide-binding domain, leucine-rich-containing family, pyrin domain-containing-3 (NLRP3, cat. no. 15101), apoptosis-associated speck-like protein containing a CARD (ASC, cat. no. 67824), procaspase-1 and caspase-1 (cat. no. 24232), tumor necrosis factor-alpha (TNF-α, cat. no. 3707), B-cell lymphoma 2 (Bcl-2, cat. no. 3498), Bcl-2-associated X protein (Bax, cat. no. 2772), cleaved caspase-3 (cat. no. 14220), AMP-activated protein kinase (AMPK, cat. no. 2532), and β-actin (cat. no. 8457) were purchased from Cell Signaling Technology (Danvers, MA, USA). Toll-like receptor 4 (TLR4, cat. no. ab217274) was obtained from Abcam (Cambridge, MA, USA). Interleukin-1beta (IL-1β, cat. no. AB1832P), phospho-AMPK at threonine 172 (p-AMPK^Thr172^, cat. no. 07-681), and peroxisome proliferator-activated receptor-γ coactivator-1alpha (PGC-1α, cat. no. ST-1203) were bought from Merck (Merck KGaA, Damstadt, Germany).

### 2.2. Animals

Twenty-six-month-old male Wistar rats, correlated to approximately 65 years of age in humans [13], were used in the study. These rats were originally obtained from Nomura Siam International (Bangkok, Thailand) and were maintained in the Laboratory Animal Unit of the Faculty of Medicine at Chiang Mai University under a 12 h light-dark cycle with standard temperature and humidity and free access to food and water. Prior to starting the experiment, all rats were physically and mentally checked by an experienced veterinarian. This included observational evaluation or physical examination to assess general condition (including body weight, skin health, coat condition, and posture) and visible signs of illness or distress. Additionally, the rats’ physical activity levels and mobility were assessed to confirm their ability to perform normal exploratory behaviors. To ensure psychological health, they were also observed for their responses to handling signs of anxiety or depression and social interactions [14]. Healthy aged animals were selected for renal functional assessment through measurements of blood urea nitrogen (BUN) and serum creatinine levels, with reference ranges of 11–25 mg/dL for BUN and 0.2–0.7 mg/dL for serum creatinine [15]. Only rats with values within these ranges were included in the study. All the study protocols were conducted in agreement with the National Research Council of Thailand’s guidelines for the use of animals and authorized by the Institutional Animal Care and Use Committee at the Faculty of Medicine, Chiang Mai University (Project number 10/2567).

### 2.3. Experimental Groups and Designs

Twenty-four male Wistar rats were randomly allocated into 4 groups (*n* = 6 each). Rats in Group 1 (Sham) and Group 2 (CLP) received vehicle (0.9% normal saline solution) via oral gavage once daily for 5 days. Thirty minutes after the final gavage, rats in Group 1 underwent a sham operation, while those in Group 2 were subjected to CLP surgery to induce sepsis. Group 3 (CLP+BBR25) and Group 4 (CLP+BBR50) were treated with BBR at a dose of 25 and 50 mg/kg, respectively, via oral gavage once daily for 5 days. On the 5th day, 30 min after the final gavage, rats were subjected to CLP surgery. The dose and administration regimen for BBR were chosen based on a study that demonstrated its potential to prevent gut-vascular barrier disruption in CLP-induced sepsis [16]. CLP surgery to induce sepsis was carried out as previously described [17]. Briefly, anesthesia was induced using 3% isoflurane inhalation (Baxter Healthcare Corporation, Deerfield, IL, USA) and maintained throughout the surgical procedure by a single intraperitoneal injection of 10 mg/kg tiletamine/zolazepam (zoletil^®^, Virbac laboratories, Carros, France). The animals were kept under a heating lamp during the operation to maintain normal body temperature. The lower abdominal area was shaved and cleaned with povidone-iodine. A midline incision of about 2–3 cm was made to access the cecum, which was then ligated with a 4/0 thread approximately 1.5 cm from the cecal tip. A sterile 21-gauge needle was used to puncture the distal cecum twice to allow a slight leakage of feces into the abdominal cavity. Before closing the abdominal cavity, 5 mL of pre-warmed sterile 0.9% normal saline solution was administered. Then, the abdomen was closed in two layers with 4-0 sutures, cleaned with povidone-iodine, and the animal was permitted to fully recover from anesthesia. For the sham operation, the rat received the same anesthetic procedure and had cecal exposure for a similar duration without ligation or perforation. All surgical techniques were performed under aseptic conditions. The entire surgical procedure took no longer than 10 min. After surgery, the rats were monitored continuously until they recovered from the effects of anesthesia and showed signs of normal activity before being returned to their cages. An additional 5 mL pre-warmed sterile 0.9% normal saline solution was administered subcutaneously once again after six hours post-surgery to maintain the central blood volume, similar to the initial treatment provided to patients in the early stages of infection.

Twenty-four hours after sepsis induction, while the animal was under thiopental anesthesia (60 mg/kg, i.p.), a blood sample was collected from the abdominal aorta to assess systemic inflammation, oxidative stress, and renal function. Both kidneys were quickly removed. One part of the kidney was immediately taken for mitochondrial studies, another piece was reserved for light and electron microscopy, and the remaining tissue was snap-frozen in liquid nitrogen and stored at −80 °C for further analyses.

### 2.4. Biochemical Analyses

#### 2.4.1. Assessments of Renal Function and Biomarkers of Kidney Injury

Blood urea nitrogen (BUN) and serum creatinine were analyzed using an automatic analyzer (Beckman Coulter, Inc., Brea, CA, USA). Serum Neutrophil Gelatinase Associated Lipocalin (NGAL), a biomarker of kidney damage, was detected by a sandwich ELISA kit (cat. no. 119602, Abcam, MA, USA) based on the manufacturer’s instruction.

#### 2.4.2. Assessments of Systemic and Renal Oxidative Stress

Systemic oxidative stress was determined by measuring the serum levels of malondialdehyde (MDA), while renal oxidative stress was assessed through the kidney tissue levels of MDA and reduced glutathione (GSH). The TBARS assay kit (cat. no. 10009055, Cayman Chemical, Ann Arbor, MI, USA) and the QuantiChrom^TM^ Glutathione Assay Kit (cat. no. DIGT-250, Bioassay Systems, Hayward, CA, USA) were used for the analyses, and the assay procedures were conducted according to the manufacturer’s guidelines.

#### 2.4.3. Assessment of Systemic Inflammation

The serum levels of tumor necrosis factor-alpha (TNF-α) were assayed using a TNF-alpha ELISA kit obtained from ABclonal Germany GmbH (cat. no. RK00029, Düsseldorf, Germany), following the manufacturer’s recommendations.

### 2.5. Histopathological Studies

#### 2.5.1. Light Microscopic Studies

The formalin-fixed kidney tissues were dehydrated in ethanol, cleared in xylene, and embedded in paraffin. Serial sections, each 4 μm thick, were deparaffinized, hydrated, and stained with hematoxylin and eosin (H&E) for examination under a light microscope by a pathologist who was unaware of the treatment protocol. Histopathological damages characterized by the presence of tubular dilatation, loss of brush border, tubular obstruction, cast formation, inflammatory infiltration, and apoptotic cells were examined. The severity of injury was graded using a semi-quantitative scoring system according to the area of tubular being affected [18] with a slight modification as followed: score 0 = no change or area of tubular injury <10%; score 1 = area of tubular injury 10–25%; score 2 = area of tubular injury 26–50%; score 3 = area of tubular injury 51–75%; and score 4 = area of tubular injury >75%.

#### 2.5.2. Electron Microscopic Studies

The electron microscopy procedure was carried out following established protocols [19]. In summary, kidney tissues were fixed overnight in 2.5% glutaraldehyde within a 0.1 M phosphate buffer (pH 7.4, at 4 °C), followed by post-fixation in 2% osmium tetroxide buffered with phosphate. Overnight fixation of kidney tissues was performed with 2.5% glutaraldehyde in a 0.1 M phosphate buffer (pH 7.4) at 4 °C, after which post-fixation was carried out in 2% osmium tetroxide buffered with phosphate. The samples were then dehydrated using a graded series of ethanol, rinsed in propylene oxide, and embedded in Epon resin with the EMbed-812 embedding kit (cat. no. EMS#14121, Electron Microscopic Sciences, Hatfield, PA, USA). Sections with a thickness of 60–80 nm were placed on copper grids, stained with uranyl acetate and lead citrate, and analyzed using a JEM-2200 FS transmission electron microscope (JEOL, Tokyo, Japan).

### 2.6. Mitochondrial Studies

#### 2.6.1. Preparations of Mitochondrial Fractions and Mitochondrial Proteins

The kidney tissue sample was first homogenized in a cold lysis buffer composed of 230 mM mannitol, 70 mM sucrose, 1 mM EDTA, and 10 mM Tris-HCl, pH 7.4. Mitochondrial isolation was achieved through differential centrifugation as previously outlined [19]. The resulting mitochondrial pellet was resuspended in a chilled respiration buffer (250 mM sucrose, 5 mM KH_2_PO_4_, 10 mM Tris-HCl, and 2 mg/mL BSA at pH 7.2). A bicinchoninic acid (BCA) assay was performed to determine mitochondrial protein concentration using bovine serum albumin as the reference standard for quantification.

#### 2.6.2. Determination of Mitochondrial Reactive Oxygen Species (ROS)

A cell-permeable fluorescent probe 2′,7′-dichlorofluorescein diacetate (DCFDA) was used to assess mitochondrial ROS production. The assay was performed according to the method previously published [19]. In brief, the mitochondrial proteins were incubated with DCFDA 2 μM for 60 min at 25 °C. Thereafter, a fluorescence microplate reader was used to measure the fluorescence emissions using excitation at 485 nm (bandwidth 10 nm) and emission at 530 nm (bandwidth 5 nm). ROS values were expressed as arbitrary units of DCF fluorescence intensity.

#### 2.6.3. Determination of Mitochondrial Membrane Potential

JC-1, a lipophilic cationic fluorescent dye, was used to assess mitochondrial membrane potential (MMP, Ψm) according to the method described in our previous study [19]. Mitochondria were stained with JC-1 at a concentration of 310 nM for 30 min at 37 °C. The green monomeric and red J-aggregated forms of JC-1 were detected by a fluorescence microplate reader at excitation/emission wavelengths of 485/530 and 485/590, respectively. Changes in MMP were reflected by changes in the red/green fluorescence intensity ratio, where a decrease in the ratio denoted MMP dissipation.

### 2.7. Western Blot Analysis

Kidney tissues were homogenized in protease inhibitor-containing lysis buffer (20 mM Tris-HCl, pH 6.8, 5 mM sodium fluoride, 1 mM sodium orthovanadate), and protein content was measured using a Quick Start™ Bradford protein assay kit (cat. no. 5000201, Bio-Rad Laboratories, Hercules, CA, USA). Following separation by 10% SDS polyacrylamide gel electrophoresis (SDS-PAGE), the proteins were loaded onto nitrocellulose membranes (Thermo Fisher Scientific, Waltham, MA, USA) and blocked with nonfat dried milk or 5% bovine serum albumin in TBST (Tris-buffered saline and Tween) buffer. After blocking, the membranes were then incubated with primary antibodies (all dilution 1:1000) against TNF-R, TLR4, pNF-κB, NLRP3, ASC, procaspase-1, caspase-1, TNF-α, IL-1β, AMPK, p-AMPK^Thr172^, PGC-1α, Bcl-2, Bax, cleaved caspase-3, and β-actin (as a loading control) at 4 °C overnight, followed by a 1 h incubation at room temperature with secondary horseradish peroxidase (HRP) antibodies (cat. no. 7074P2, Cell Signaling Technology, Danvers, MA, USA). Clarity ECL Western blotting substrate (cat. no. 1705061, Bio-Rad Laboratories, Hercules, CA, USA) was used to detect protein bands, and the Image J program version 1.54d (National Institute of Health, Bethesda, MD, USA) was used to quantify the levels of protein expression.

### 2.8. Statistical Analysis

Results are presented as means ± SEM. Individual data points are shown as dot plots. Each point represents the average from multiple independent analyses, with three biological replicates per group, and technical replicates for each biological replicate. For statistical evaluation, a one-way analysis of variance (ANOVA) followed by Tukey’s post hoc test was performed using the SPSS version 25 program (IBM Corporation, Armonk, NY, USA). The significance level was set at *p* < 0.05.

## 3. Results

### 3.1. BBR Reduces Sepsis-Induced Systemic Oxidative Stress and Inflammation

**Figure 1:**
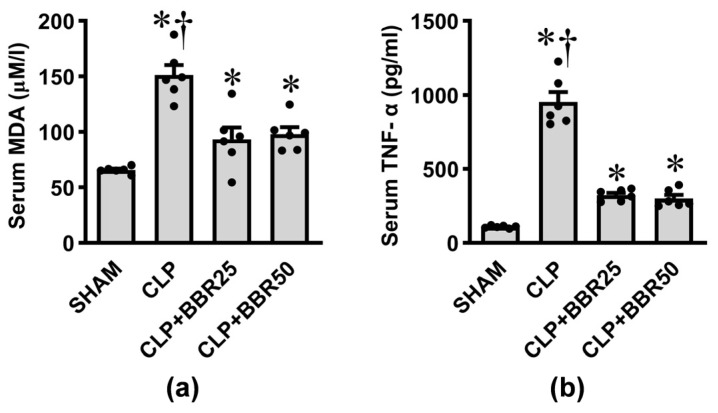
Effects of CLP induction and BBR treatment on serum levels of (**a**) malondialdehyde (MDA) and (**b**) tumor necrosis factor-alpha (TNF-α). Values are means ± SEM (*n* = 6 each). Each data point represents the average of three biological replicates. SHAM: rats treated with vehicle and underwent sham operation; CLP: rats treated with vehicle and underwent CLP induction; CLP+BBR25 and CLP+BBR50: rats treated with BBR 25 and 50 mg/kg, respectively, and underwent CLP induction. * *p* < 0.05 vs. SHAM, ^†^ *p* < 0.05 vs. CLP+BBR.

The levels of serum MDA and TNF-α were used to evaluate sepsis-induced systemic oxidative stress and inflammatory response following CLP incidence. As shown in Figure 1a,b, all the groups subjected to CLP induction showed significant increases in the serum MDA and TNF-α levels compared to the sham group (all *p* < 0.05). Interestingly, pretreatment of rats with BBR significantly reduced serum MDA and TNF-α, although the levels of both markers did not fully recover to baseline controls (all *p* < 0.05). In addition, no dose-related responses were observed between the two BBR doses used in this study.

### 3.2. BBR Mitigates Sepsis-Induced AKI

**Figure 2:**
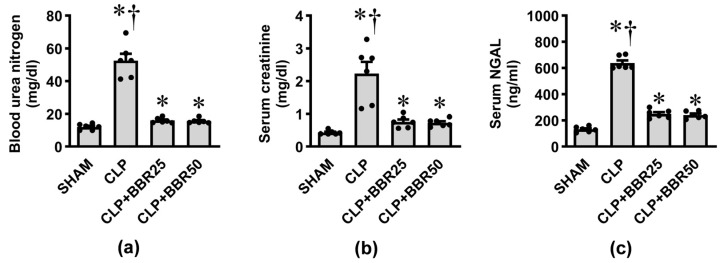
Effects of CLP induction and BBR treatment on renal function: (**a**) blood urea nitrogen; (**b**) serum creatinine; and (**c**) serum NGAL. Values are means ± SEM (*n* = 6 each). Each data point represents the average of three biological replicates. SHAM: rats treated with vehicle and underwent sham operation; CLP: rats treated with vehicle and underwent CLP induction; CLP+BBR25 and CLP+BBR50: rats treated with BBR 25 and 50 mg/kg, respectively, and underwent CLP induction. * *p* < 0.05 vs. SHAM, ^†^ *p* < 0.05 vs. CLP+BBR.

To evaluate the effect of CLP-induced sepsis on renal function, the levels of BUN, serum creatinine, and serum NGAL were determined (Figure 2). In the CLP group, about a five-fold increase in BUN (Figure 2a), serum creatinine (Figure 2b), and serum NGAL (Figure 2c) levels were evident in comparison with the sham group (all *p* < 0.05). Treatment with BBR prior to CLP induction, regardless of the dose, markedly blunted the rise in these parameters (all *p* < 0.05).

### 3.3. BBR Diminishes Sepsis-Induced Kidney Histopathological Damages

**Figure 3:**
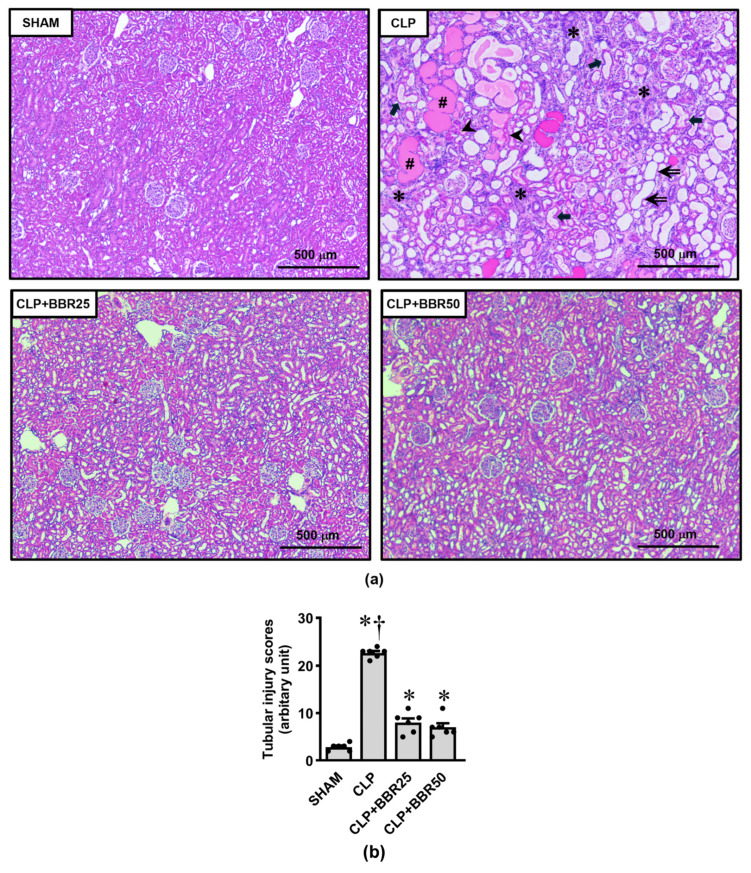
Effects of CLP induction and BBR treatment on renal histopathological changes; (**a**) kidney sections stained with hematoxylin and eosin (H&E, 4× magnification); (**b**) tubular injury score. Values are means ± SEM (*n* = 6 each). Each data point represents the average of three biological replicates. SHAM: rats treated with vehicle and underwent sham operation; CLP: rats treated with vehicle and underwent CLP induction; CLP+BBR25 and CLP+BBR50: rats treated with BBR 25 and 50 mg/kg, respectively, and underwent CLP induction. * *p* < 0.05 vs. SHAM, ^†^ *p* < 0.05 vs. CLP+BBR. The double arrow indicates tubular dilatation with brush border loss; the block arrow denotes tubular obstruction; the hash sign (#) shows hyaline casts; the asterisk (*) highlights inflammatory infiltration; and the arrowhead points to apoptotic cells.

H&E-stained kidney tissues from each of the groups under investigation are displayed in Figure 3a. The kidney section of the sham-operated rat showed typical histomorphology. In contrast, the CLP group exhibited a profound and diffuse range of abnormalities that were consistent with their renal functional changes, including tubular dilation, loss of brush border, tubular obstruction, hyaline cast formation, massive inflammatory cell infiltration, and pyknotic nuclei with apoptosis and/or necrosis. The lesions occupied almost all the interstitial area. Upon pretreatment with either a low or high dose of BBR, these changes were noticeably reduced. Semi-quantitative analysis of the renal tubular damages (Figure 3b) provided further support to this view, as indicated by significant reductions in the tubular injury scores in the BBR-treated CLP groups compared to that of the untreated CLP group (all *p* < 0.05).

### 3.4. BBR Attenuates Sepsis-Induced Renal Oxidative Stress

**Figure 4:**
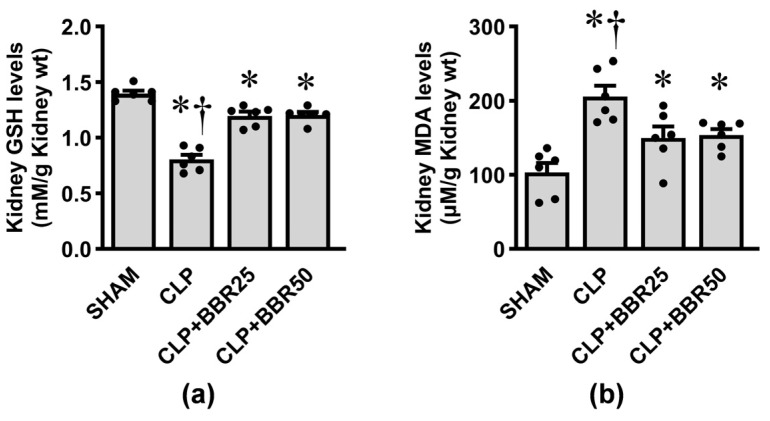
Effects of CLP induction and BBR treatment on kidney tissue levels of (**a**) reduced glutathione (GSH) and (**b**) malondialdehyde (MDA). Values are means ± SEM (*n* = 6 each). Each data point represents the average of three biological replicates. SHAM: rats treated with vehicle and underwent sham operation; CLP: rats treated with vehicle and underwent CLP induction; CLP+BBR25 and CLP+BBR50: rats treated with BBR 25 and 50 mg/kg, respectively, and underwent CLP induction. * *p* < 0.05 vs. SHAM, ^†^ *p* < 0.05 vs. CLP+BBR.

To evaluate the impact of CLP-induced sepsis on renal oxidative status, the changes in antioxidant GSH and lipid peroxidation product MDA in kidney tissue were measured. As expected, a considerable reduction in GSH levels (Figure 4a) and a substantial elevation in MDA levels (Figure 4b) were observed after CLP induction compared to those in the sham group (all *p* < 0.05). Notably, these changes were significantly attenuated by BBR pretreatment (all *p* < 0.05).

### 3.5. BBR Mitigates Sepsis-Induced Kidney Mitochondrial Damages

**Figure 5:**
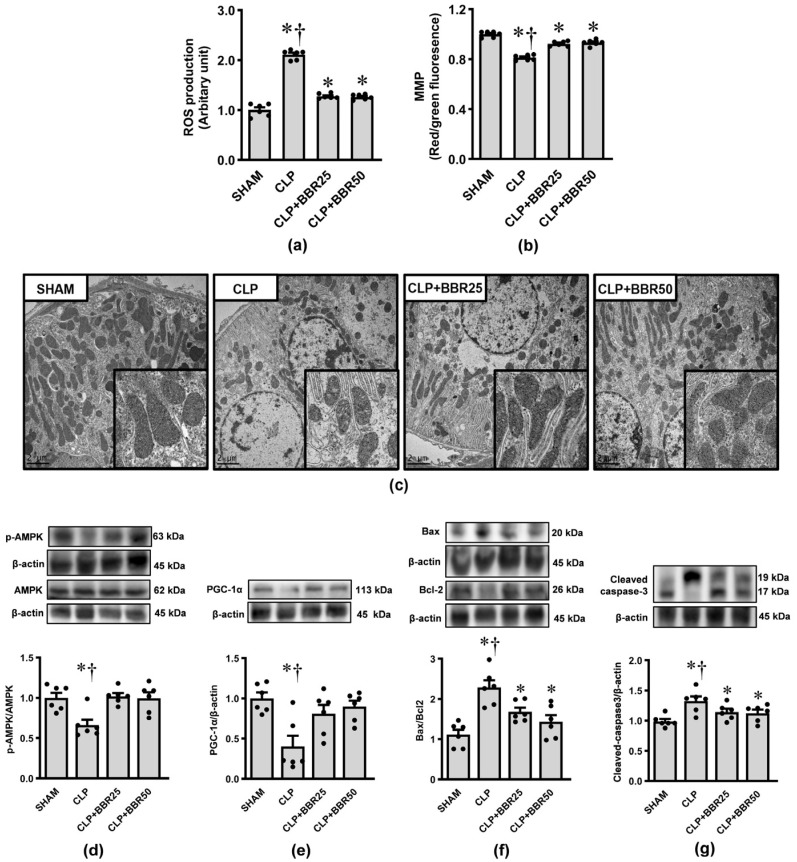
Effects of CLP induction and BBR treatment on kidney mitochondrial function, ultrastructure, and the expressions of proteins related to cellular apoptosis; (**a**) mitochondrial reactive oxygen species (ROS) production; (**b**) mitochondrial membrane potential changes (MMP); (**c**) transmission electron microscopic images of proximal tubules (original magnification: 3000×); (**d**) pAMPK/AMPK; (**e**) PGC-1α; (**f**) Bax/Bcl-2; and (**g**) cleaved caspase-3. β-actin was used as a loading control. Values are means ± SEM (*n* = 6 each). Each data point in (**a**,**b**) represents the average of three biological replicates. SHAM: rats treated with vehicle and underwent sham operation; CLP: rats treated with vehicle and underwent CLP induction; CLP+BBR25 and CLP+BBR50: rats treated with BBR 25 and 50 mg/kg, respectively, and underwent CLP induction. * *p* < 0.05 vs. SHAM, ^†^ *p* < 0.05 vs. CLP+BBR.

Figure 5 demonstrates the effects of CLP induction and BBR treatment on kidney mitochondrial function, ultrastructure, and key proteins involved in maintaining mitochondrial homeostasis. Mitochondrial ROS production (Figure 5a) was markedly and significantly increased, whereas mitochondrial membrane potential (Figure 5b) was significantly decreased in the CLP group compared to those of the sham group (all *p* < 0.05). Similarly, electron microscopic images (Figure 5c) revealed CLP-induced ultrastructural changes in mitochondria, i.e., mitochondrial swelling, fragmentation, less dense cristae, and considerable reduction in mitochondrial number. Induction of CLP also produced significant changes in the expression levels of proteins related to mitochondrial metabolism, biogenesis, and bioenergetics, as shown by significantly decreased pAMPK/AMPK as well as PGC-1α (Figure 5d,e). Increased Bax/Bcl-2 (Figure 5f) and cleaved caspase-3 (Figure 5g), which were involved in apoptosis, were also detected. Regardless of dosage, BBR therapy effectively decreased all the changes brought on by CLP (all *p* < 0.05).

### 3.6. BBR Inhibits Sepsis-Induced TLR4/NF-κB and NLRP3 Inflammasome Activations

**Figure 6:**
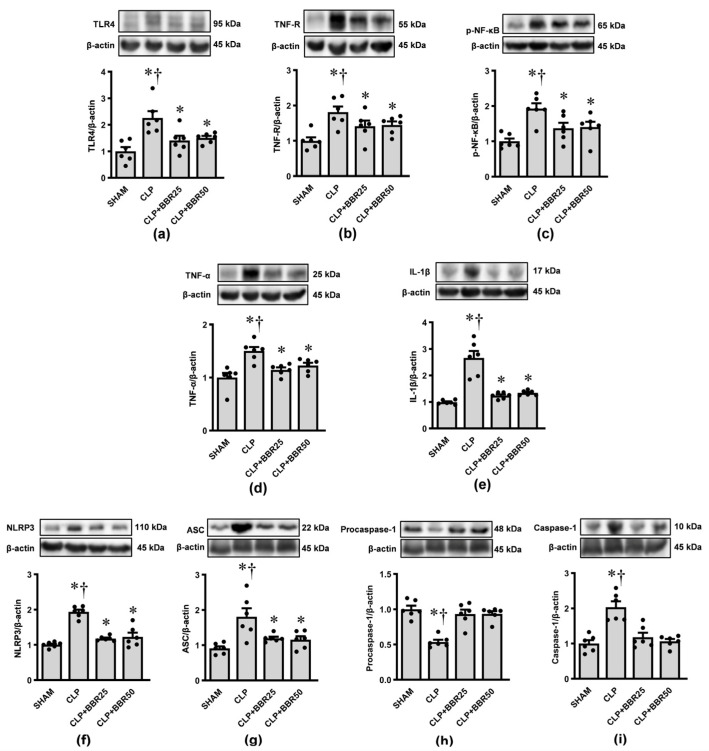
Effects of CLP induction and BBR treatment on renal cortical expression of (**a**) TLR4; (**b**) TNF-R; (**c**) pNF-κB; (**d**) TNF-α; (**e**) IL-1β; (**f**) NLRP3; (**g**) ASC; (**h**) procaspase-1; and (**i**) caspase-1. β-actin was used as a loading control. Values are means ± SEM (*n* = 6 each). SHAM: rats treated with vehicle and underwent sham operation; CLP: rats treated with vehicle and underwent CLP induction; CLP+BBR25 and CLP+BBR50: rats treated with BBR 25 and 50 mg/kg, respectively, and underwent CLP induction. * *p* < 0.05 vs. SHAM, ^†^ *p* < 0.05 vs. CLP+BBR.

To investigate whether BBR affects CLP-induced septic AKI through the activation of the TLR4/NF-κB and NLRP3 inflammasome pathways, we performed Western blot analysis to measure the expression levels of key proteins involved in these pathways. The results are shown in Figure 6. Quantitative analysis revealed that CLP induction significantly increased the protein levels of TLR4, TNF-R, pNF-κB, TNF-α, IL-1β, NLRP3, ASC, and caspase-1, while significantly decreasing procaspase-1, compared to the levels in rats that underwent only sham operation (all *p* < 0.05). Administration of BBR to rats prior to CLP significantly diminished these changes (all *p* < 0.05). Additionally, no dose-dependent effects were observed between the BBR-treated groups.

## 4. Discussion

This study underscores the critical importance of developing effective treatments for SA-AKI in elderly patients. Our findings provide novel evidence to show BBR as a promising therapeutic strategy for SA-AKI in the elderly. The ability of BBR to target several pathways implicated in renal inflammation, oxidative stress, and mitochondrial dysfunction suggests that it holds potential to improve SA-AKI outcomes for this vulnerable population.

Sepsis is a major healthcare concern, especially for the elderly population. Over 60% of sepsis cases occur in patients over 65 years old, with both sepsis-related complications and mortality rates increasing significantly with age [20]. However, much of the basic research on sepsis has been conducted on young animals, usually less than 3 months old. This age is roughly equivalent to a human under 20 years old. As a result, the study did not correspond to the unique pathophysiology of sepsis in the elderly, rendering it unsuitable for clinical application [20]. Although a D-galactose-induced aging rodent model has been developed and used to study age-associated diseases, it does not fully replicate natural human aging. This is because the degree of D-galactose-induced aging varies depending on both dosage and duration. Accordingly, the relationship between D-galactose-induced aging and natural aging remains unclear. Moreover, it is not feasible to accurately determine the exact age of animals subjected to aging with D-galactose [20]. In this investigation, we used a natural aging rat at 26 months of age, which equates to about 65 years of human life [13] as the study model. Therefore, information obtained from our aged rat is realistic and able to provide useful information for elderly patients.

Another significant point to consider is the sepsis model, as improper use of the model could limit clinical applicability. The lipopolysaccharide (LPS) model and the cecal ligation and puncture (CLP) model are both commonly used in preclinical research to study sepsis and its complications. In our study, we selected the CLP model not only because it has been recognized as the gold-standard model of sepsis research [17,21,22], but also because it outperformed the LPS model. The CLP model simulates sepsis in a more clinically meaningful manner by causing polymicrobial peritonitis, which closely resembles the complicated pathophysiology of human sepsis. This model accounts for the complex relationships between systemic infection, inflammatory responses, and organ dysfunction [21,23,24]. In contrast, no active infection occurred in the LPS model because the experimental animal was directly administered endotoxin. In this model, LPS creates an endotoxemic response without actual microbial infection, which may oversimplify disease dynamics in human sepsis and cause variability in research outcomes [25]. Thus, our study model of CLP-induced sepsis in naturally aged rats is appropriate and allows us to examine SA-AKI and the protective potential of BBR in a more comprehensive and realistic setting.

In the present study, CLP induction in aged rats led to an acute deterioration of renal function, as indicated by significant increases in BUN, serum creatinine, and serum NGAL, a sensitive biomarker for AKI, compared with aged-match sham controls, suggesting the development of SA-AKI. Histopathological findings of extensive tubular damage with massive inflammatory infiltration occupying almost the entire renal interstitial area further corroborated the acute and inflammatory nature of kidney injury in our experimental model.

SA-AKI is a severe complication of sepsis resulting from a dysregulated host response to an infective insult. The innate immune system is the host’s first line of defense against infection, utilizing pattern recognition receptors (PRRs) to detect pathogen-associated molecular patterns (PAMPs) and damage-associated molecular patterns (DAMPs) [26]. PAMPs are exogenous microbial products, while DAMPs are endogenous molecules released from stressed, damaged, or dying cells. Both PAMPs and DAMPs can trigger and enhance inflammatory responses through pattern recognition receptors such as TLR4 and NLRP3 [26]. Although we did not directly measure PAMPs and DAMPs in blood circulation, our study model as well as our findings provide compelling evidence supporting their involvement through indirect markers and downstream signaling pathways. The CLP model inherently induces the release of PAMPs due to its mechanism of inducing sepsis through gut flora translocation [17,21]. This process generates significant oxidative stress and inflammatory responses, which is corroborated by our observation of increased serum levels of MDA and TNF-α. MDA is a by-product of lipid peroxidation, a process that occurs during oxidative stress and cellular damage. Elevated serum MDA levels indicate not only oxidative damage but also a role for MDA as a DAMP, which may contribute to the inflammatory response by signaling tissue damage and activating immune cells to cause further inflammation. Moreover, elevated serum TNF-α levels support a systemic inflammatory response, consistent with activation of pro-inflammatory pathways mediated by PAMPs and DAMPs.

Our Western blot analysis revealed increased levels of TLR4 and pNF-κB, alongside elevated TNF-α and IL-1β in the kidney tissue of CLP-induced septic rats. Notably, activation of the NLRP3 inflammasome complex, including ASC, caspase-1, and caspase-1-dependent IL-1β production, was also observed post-CLP. These findings underscore the role of TLR4-mediated signaling pathways in the initiation and progression of inflammation during SA-AKI. TLR4 is recognized for its ability to detect various PAMPs and DAMPs, which activate NF-κB and lead to the production of pro-inflammatory cytokines, including TNF-α and IL-1β [27,28]. Regarding the NLRP3 inflammasome, its activation occurs in response to multiple cellular stressors and tissue damage, facilitating the release of pro-inflammatory cytokines such as IL-1β [29,30]. The increased activation of the NLRP3 inflammasome suggests a significant contribution from DAMPs released by damaged tissues in the inflammatory response following CLP-induced SA-AKI. Additionally, our findings highlight the role of TNF-α and non-pattern recognition receptor (non-PPR) mechanisms in mediating inflammation and exacerbating renal injury after CLP. We detected an increased expression of TNF receptors (TNF-R) in kidney tissue following CLP incidence. This implies an active role for TNF-α in the pathological processes of SA-AKI and emphasizes the relevance of non-pattern recognition receptor mechanisms in this context. Taken together, our data suggested that while PAMPs and DAMPs are integral to the inflammatory milieu via pattern recognition receptor activation, TNF-α-mediated signaling through its receptors may further worsen kidney injury independent of these mechanisms.

Of particular significance, our study demonstrated that administration of BBR prior to CLP induction, regardless of the dose, effectively mitigated SA-AKI in aged rats. BBR, a natural isoquinoline alkaloid found in various medicinal plants, has attracted considerable attention due to its broad-spectrum pharmacological properties. It is renowned for its potent anti-inflammatory, antioxidant, anti-apoptotic, and antimicrobial activities, making it a promising therapeutic agent for a range of conditions, including kidney diseases [6,7,8,9]. In our study, BBR pretreatment notably alleviated sepsis-induced renal dysfunction and abnormal histological features. This improvement was evidenced by significant reductions in serum levels of BUN, creatinine, and NGAL, together with remarkable preservation of tubular structural integrity. Additionally, these outcomes were associated with a decrease in systemic oxidative stress and inflammation, indicated by lower serum levels of MDA and TNF-α. Given the recognized properties of BBR, it is plausible that it counteracts polymicrobial organisms released from the gut barrier during the early phase of sepsis, thus protecting against oxidative stress (reflected in our study by serum MDA) and subsequent attenuation of the inflammatory cascade. We also observed decreased activation of both pattern recognition receptors (TLR4/NF-κB and NLRP3 inflammasome signaling pathways) and non-pattern recognition receptors (TNF-α/TNF-R signaling) following BBR pretreatment. These findings further support the notion that BBR exerts protective effects against SA-AKI by inhibiting mechanisms associated with both pattern and non-pattern recognition receptors.

Mitochondria are a major source of ROS production within the body. Mitochondrial dysfunction and subsequent oxidative stress generation play a pivotal role in the development and progression of AKI following sepsis [23,31,32]. Our study revealed that mitochondrial function is compromised upon CLP induction, as evidenced by dissipation of the mitochondrial membrane potential (ΔΨm) and an increase in mitochondrial ROS production, which signifies impaired mitochondrial integrity and bioenergetic failure [31]. The irregularity of mitochondrial ultrastructure and the reduction in mitochondrial number observed from the transmission electron micrograph provided further support for mitochondrial impairment. These alterations involved an upregulation of the pro-apoptotic protein Bax, a downregulation of the anti-apoptotic protein Bcl-2, and an increased expression of cleaved caspase-3, a key regulator of apoptosis. Together, these changes indicate an imbalance in apoptotic signaling pathways that promotes cell death in our study. This sequence of events leads to significant renal oxidative stress and subsequent kidney damage following CLP, as indicated by increased levels of MDA and decreased GSH in the kidney tissues. Remarkably, pretreatment with BBR effectively mitigated all these changes caused by sepsis. Our findings align with previous studies that have documented BBR’s antioxidant properties and, particularly, mitochondrial protective abilities in various disease models [8,33,34]. In addition, we detected that the expressions of p-AMPK/AMPK and PGC-1α were significantly reduced in the CLP group compared to the sham group. Treatment with BBR was able to restore these changes. The findings further support our view that BBR offers protective effects by reversing mitochondrial dysfunction caused by sepsis. The AMPK plays a pivotal role in cellular energy regulation by promoting mitochondrial biogenesis through the activation of PGC-1α. PGC-1α acts as a master regulator of mitochondrial biogenesis, enhancing the expression of genes responsible for mitochondrial function, oxidative phosphorylation, and energy metabolism [35]. In the context of SA-AKI, the reduction in p-AMPK and PGC-1α observed in the CLP group indicates impaired mitochondrial biogenesis, which contributes to mitochondrial dysfunction, oxidative stress, and energy depletion. BBR also helps reset the balance between mitochondrial biogenesis and degradation, allowing cells to better cope with the energetic and oxidative stress caused by sepsis. This resetting of mitochondrial homeostasis may enhance the cell’s ability to recover from injury and maintain essential bioenergetic functions.

SA-AKI is a complex condition driven by a cascade of interconnected pathological mechanisms, including oxidative stress, mitochondrial dysfunction, and inflammation. Understanding the interplay between these factors is critical to contextualizing the protective effects of BBR in our study. Oxidative stress plays a central role in SA-AKI by generating excessive reactive oxygen species (ROS), which leads to cellular and mitochondrial damage. Mitochondria are both a source and target of ROS, and mitochondrial dysfunction exacerbates ROS production, creating a vicious cycle that promotes further damage. In this context, mitochondrial dysfunction impairs essential bioenergetic processes, such as ATP production, and leads to structural damage, ultimately compromising cell viability in the kidney. This mitochondrial damage also contributes to the activation of key inflammatory pathways, including the TLR4/NF-κB and NLRP3 inflammasome pathways, which are known to drive inflammatory responses in SA-AKI. Excessive ROS not only damages cellular structures but also acts as a signaling molecule that triggers these inflammatory pathways, further amplifying the inflammatory response. Inflammatory mediators, in turn, worsen mitochondrial damage, forming a self-perpetuating cycle of oxidative stress, mitochondrial dysfunction, and inflammation that accelerates kidney injury. Our findings demonstrate that BBR breaks this pathological cycle by both preserving mitochondrial integrity and reducing ROS production, thus preventing the activation of these inflammatory pathways. By mitigating oxidative stress, BBR protects mitochondrial function and, in doing so, reduces the downstream inflammatory responses that contribute to SA-AKI. By elucidating this relationship between oxidative stress, mitochondrial dysfunction, and inflammation, our study provides a clearer understanding of how BBR exerts its protective effects in SA-AKI.

While the management of SA-AKI typically involves supportive care such as fluid resuscitation, vasopressor therapy, and renal replacement therapy (RRT), these treatments primarily address the symptoms of organ failure without directly targeting the underlying pathophysiological mechanisms that lead to kidney damage [36]. Antioxidant therapies such as N-acetylcysteine (NAC) have been explored in the context of SA-AKI to reduce oxidative stress, but their clinical efficacy has been limited [37]. The novelty of BBR lies in its ability to modulate key molecular pathways involved in mitochondrial dysfunction, oxidative stress, and inflammation, which are central to the progression of SA-AKI. Future studies could explore the potential of combining BBR with standard therapies, such as RRT and vasopressors, to investigate whether this combined approach could offer synergistic benefits in the clinical management of SA-AKI.

While our study demonstrates that BBR mitigates SA-AKI in aged rats through the preservation of mitochondrial integrity and inhibition of inflammatory pathways, there are several limitations that should be acknowledged. First, although pathogen-associated molecular patterns (PAMPs) and damage-associated molecular patterns (DAMPs) are key triggers in sepsis-induced inflammation, we did not directly measure these factors in the current study. Future research should aim to evaluate the role of PAMPs and DAMPs in BBR’s protective effects against SA-AKI to better elucidate the underlying mechanisms. Additionally, potential sex differences were not addressed, as only male rats were included in this study. Evidence suggests that there may be significant sex differences in both the pathophysiology of sepsis and kidney injury, as well as in the response to treatments like BBR [38,39]. For example, female animals often show different immune responses and injury patterns in sepsis models, which may be influenced by hormonal and genetic factors. The exclusion of female rats limits the generalizability of our findings. Future studies should incorporate both male and female subjects to explore potential sex-specific effects of BBR, particularly in the context of SA-AKI. While we focused on aged rats in this study due to the increased risk of AKI in elderly populations, comparisons between young and aged animals could provide additional insights into age-related differences in the renoprotective effects of BBR. Lastly, although we observed a protective effect at both 25 mg/kg and 50 mg/kg doses of BBR, we did not find a clear dose–response relationship. One possible explanation is that the higher dose of BBR (50 mg/kg) may have reached a plateau in its efficacy, suggesting that 25 mg/kg was sufficient to achieve the maximal therapeutic effect in this model. Another consideration could be the pharmacokinetics of BBR, as it has relatively low bioavailability due to extensive first-pass metabolism, which could limit the additional benefits of increasing the dose beyond 25 mg/kg. Additionally, BBR’s effects on mitochondrial protection and inflammation might exhibit a threshold response rather than a linear dose–response relationship, where the biological systems reach saturation at a certain concentration. To address this limitation in future studies, it would be valuable to explore a wider range of doses, including both lower and higher concentrations, to better characterize the dose–response relationship. Additionally, pharmacokinetic studies should be integrated to assess the absorption, distribution, and elimination of BBR at different doses. Investigating potential biomarkers of BBR activity may also help determine whether the therapeutic window is dose-limited. However, the overall results obtained in our study suggest that BBR’s antioxidative properties are pivotal in reducing oxidative injury and maintaining mitochondrial integrity, which in turn helps prevent renal apoptosis and control inflammatory responses in SA-AKI.

## 5. Conclusions

Our study reveals that BBR offers significant protection against SA-AKI through its effects on oxidative stress, apoptosis, and inflammatory cytokine production. The mechanisms of BBR include the inhibition of TLR4/NF-κB signaling and the NLRP3 inflammasome pathway, along with the preservation of mitochondrial integrity. These findings underscore the potential of BBR as a therapeutic agent for SA-AKI in the elderly and provide a basis for its clinical evaluation. Future research should focus on translating these promising preclinical results of BBR into clinical trials to evaluate its safety and efficacy in human populations facing this challenging condition.

## Data Availability

The data presented in this study are available from the corresponding author on reasonable request.
